# Identification of Ocular and Auditory Manifestations of Congenital Rubella Syndrome in Mbingo

**DOI:** 10.1155/2014/981312

**Published:** 2014-11-25

**Authors:** Imran Jivraj, Chris J. Rudnisky, Emmanuel Tambe, Graham Tipple, Matthew T. S. Tennant

**Affiliations:** ^1^Department of Ophthalmology, University of Alberta, 10240 Kingsway Avenue NW, Edmonton, AB, Canada T5H 3V9; ^2^Department of Ophthalmology, Mbingo Baptist Hospital, Cameroon; ^3^National Microbiology Laboratory, Public Health Agency of Canada, Canada

## Abstract

*Purpose*. Congenital rubella syndrome (CRS) is a global cause of preventable hearing impairment, blindness, and intellectual impairment. The present study sought to identify ocular and auditory manifestations of CRS in school-aged children in Mbingo, Cameroon. *Design*. Cross sectional study. *Subjects*. Students at two schools, one for children with hearing impairment, were screened for cataract, congenital glaucoma, and pigmentary retinopathy. *Methods*. Students underwent seven-field digital fundus photography through a dilated pupil using a Topcon NW200 nonmydriatic camera. Images were assessed by retina specialists in Canada via teleophthalmology. Clinical evidence was integrated to form case definitions for CRS based on Center for Disease Control and Prevention guidelines. Serological evidence of rubella infection was obtained using standardized IgG antibody titers. *Main Outcome Measure*. Number of probable and suspicious cases of CRS. *Results*. Between September 2009 and May 2010, 320 students participated. There were 28 (10.2%) probable cases, 104 (37.8%) suspects, and 143 (52.0%) unaffected. Rubella IgG serology was positive in 79 (48.7%) of children with hearing impairment and 11 (7.4%) of children with normal hearing. *Conclusions*. The present study identified 28 probable cases of CRS. Furthermore, 92.6% of students with normal hearing did not possess rubella IgG antibodies making future cases of CRS likely without intervention.

## 1. Introduction

Congenital rubella syndrome (CRS) is a major global cause of preventable hearing impairment, blindness, and intellectual impairment [1]. Worldwide, it is estimated that approximately 100,000 infants are born with CRS each year; in countries with effective vaccination programs, new cases are uncommon [2]. As of 2012, 132 of 194 World Health Organization member countries, including three African countries, had incorporated the rubella vaccine into their national childhood immunization program, which represented a 33% increase from 99 member states in 2000 [2–4]. In 2013, Rwanda became the first country in sub-Saharan Africa to offer the rubella vaccination as part of their national immunization strategy, with plans for Ghana and Senegal to follow [5]. A single epidemiological study has estimated the seroprevalence of rubella antibodies in pregnant women in Yaounde, Cameroon, to be 83.9%, suggesting that significant* in utero *vulnerability exists in women who have never been exposed to the virus [6].

**Figure 1:**
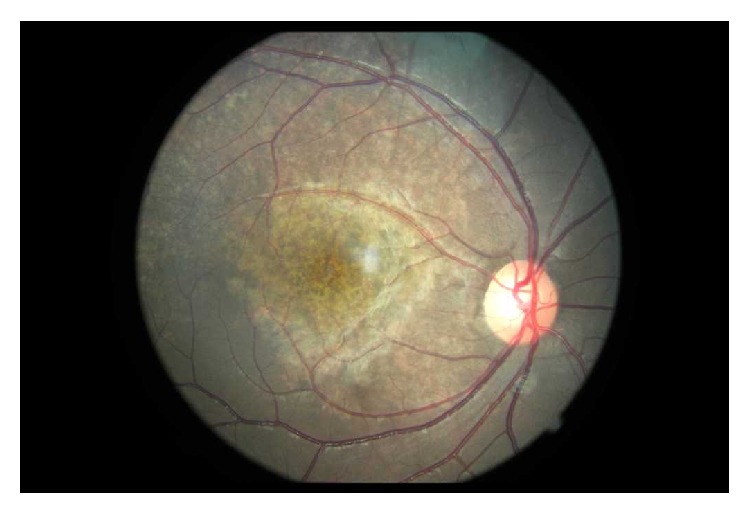
Salt and pepper retinopathy at the posterior pole in congenital rubella syndrome.

Maternal rubella infection in pregnancy may lead to a spectrum of fetal outcomes: an infant may be born normally, with congenital abnormalities, or spontaneously abort. Congenital rubella syndrome (CRS) denotes the presence of any combination of classic features which may accompany rubella infection early in gestation. As many as 50% of infants with CRS may appear normal at birth but will develop features of CRS afterwards [7]. The constellation of clinical features of CRS consists of sensorineural hearing impairment, intellectual impairment, cardiac defects, and ocular findings [8]. Deafness is the most common abnormality seen in CRS, and the proportion of children who have hearing loss may be as high as 70% [9]. Of the ocular defects, a “salt and pepper” retinopathy is most commonly observed and is seen in 40–60% of cases but does not typically have visual consequences unless neovascularization develops [9]. The retinopathy is varied in appearance and distribution and is most commonly located in the posterior pole (see Figure 1).

Nuclear cataracts occur in about one-third of all cases and are bilateral in 50% [7, 8]. Other diverse but less common abnormalities have been reported [10–12]. Maternal rubella infection at different points in the pregnancy will lead to different rates of fetal infection and combinations of classic CRS features (see Table 1) [7].

The primary purpose of this study was to identify cases of CRS with ocular or auditory involvement in children attending two schools, one for students with normal hearing and the other for students with hearing impairment in Mbingo, Cameroon, using teleophthalmology. Secondary outcomes included identifying the seroprevalence of rubella IgG antibodies and the presence of salt and pepper retinopathy.

## 2. Methods 

Students at two schools in Mbingo, Cameroon, were screened for clinical evidence of congenital rubella syndrome. The two schools were chosen due to their proximity to the town of Mbingo. One of the schools was for children with normal hearing and the second school was for children with hearing impairment. A teleophthalmology partnership between the University of Alberta and the Mbingo Hospital enabled transmission of fundus photographs to retina specialists in Edmonton, Alberta, for confirmation of the diagnosis of rubella retinopathy. Research ethics approval was granted through both the Cameroon Baptist Convention Institutional Review Board and the University of Alberta Research Ethics Board. This research adhered to the tenets of the Declaration of Helsinki.

Patients underwent seven-field digital photography of the retina through a dilated pupil using a Topcon NW200 nonmydriatic retinal camera. Images of the anterior segment, optic disc, and macula were captured. Original Joint Photographic Experts Group (JPEG) images were saved (1800 × 1200, 2.2 million pixels) and stored onto an attached laptop computer. The images were then further JPEG compressed 22 : 1 to facilitate transmission via a satellite connection with limited bandwidth. Digital images were then linked to Extendable Markup Language (XML) metadata as a single encrypted file and transmitted via the internet to a secure, web-based server (https://teleophthalmology.com/; Secure Diagnostic Imaging Ltd., Edmonton, Alberta, Canada) for review by Canadian-based ophthalmologists at the Royal Alexandra Hospital Teleophthalmology Reading Centre. Images were viewed on a dual-monitor system through liquid crystal display shutter goggles (Stereographics CrystalEyes Wired, Stereographics, San Rafael, CA) using proprietary 3D viewing software (SDI Stereoviewer, Secure Diagnostic Imaging, Edmonton, Alberta, Canada) and an accompanying online ETDRS grading template.

Serological evidence of rubella infection was obtained using standardized rubella IgG antibody titers analyzed at a local medical laboratory in Mbingo, Cameroon. Rubella IgG antibodies were detected using the human rubella IgG ELISA Test Kit (Diagnostic Automation, http://www.rapidtest.com/) which offers excellent sensitivity (100%) and specificity (100%) according to the manufacturer. The positive cutoff was >15 IU/mL and antibody titers less than this were considered negative. IgM levels were not measured as the sample population would no longer demonstrate positive IgM levels from CRS due to age. Some participants were unavailable for retinal photographs or blood testing and therefore not all participants had complete testing records.

### 2.1. Case Definitions

Clinical evidence was integrated to form two case definitions for CRS based on Center for Disease Control (CDC) and Prevention guidelines, 2012 [13–15]. Systemic sequelae of CRS used in the CDC guidelines were not assessed including congenital heart disease, purpura, hepatosplenomegaly, jaundice, microcephaly, or developmental delay. Thus, clinical criteria for case definitions were restricted to the detection of cataract, congenital glaucoma, pigmentary retinopathy, and hearing impairment.

For the purposes of this study a* CRS suspect* was defined as an individual with one of cataract, congenital glaucoma, pigmentary retinopathy, or hearing impairment. A* probable case* of CRS was defined as an individual with two or more of cataract, congenital glaucoma, pigmentary retinopathy, or hearing impairment. Unaffected children demonstrated none of the above findings. Because serological testing could not be performed according to the Center for Disease Control guidelines, no cases of CRS could be confirmed.

## 3. Results

Between September 2009 and May 2010, all 320 students from two schools in Mbingo, Cameroon, participated in this study. The average age of students from both schools was 11.8 ± 2.8 years and 58.2% of students were male. Fundus photographs were available for 275 (85.9%) participants and rubella IgG serology was available for 310 (96.9%) patients. Complete records including fundus photographs and serology were available for 268 (83.8%) participants.

### 3.1. Rubella Serology Status

**Figure 2:**
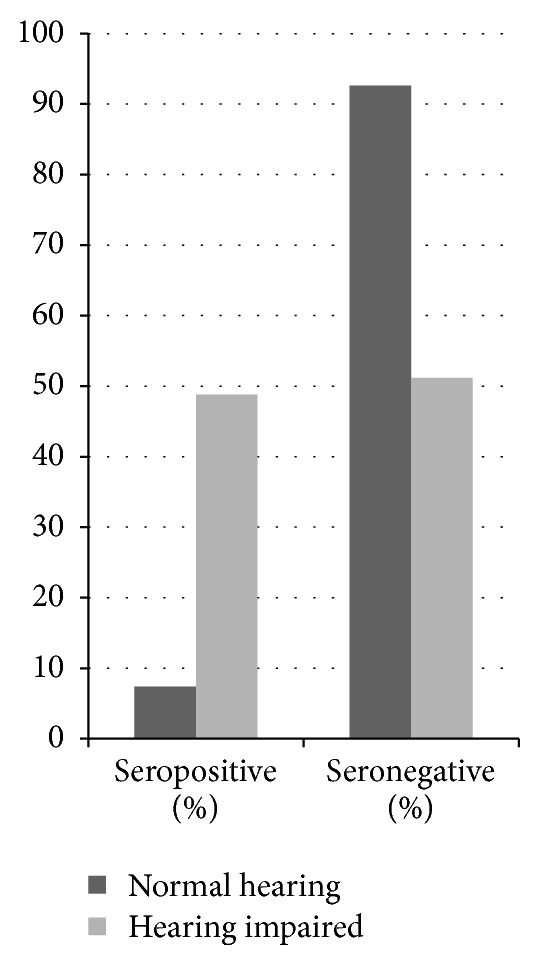
Rates of rubella IgG seropositivity among children with and without hearing impairment in Northwest Cameroon. Children with hearing impairment were significantly more likely to have positive serology (*P* < 0.0001), and children with normal hearing were significantly more likely to have negative serology (*P* < 0.0001).

Serological investigations for rubella were carried out at a local laboratory in Mbingo. Of the 310 subjects with complete serology, there were 162 students with hearing impairment, with a mean age of 13.0 ± 3.0 years, and 148 students with normal hearing, with a mean age of 10.5 ± 1.8 years. The students with hearing impairment were significantly older than those without hearing impairment in the study population (*P* < 0.0001). Ninety students (29.0%) were positive for rubella IgG antibodies and 220 (71.0%) were negative. Hearing impaired children were seven times more likely to have positive serology (48.8%) than children with normal hearing (7.4%; *P* < 0.0001). Conversely, children with normal hearing were almost two times more likely to have negative serology (92.6%) than hearing impaired children (51.2%; *P* < 0.0001) (see Figure 2).

### 3.2. Ocular Manifestations of Rubella

Fundus photographs were obtained for 549 eyes of 275 (85.9%) students. 58 (10.5%) eyes of 29 patients showed clear evidence of rubella retinopathy. Twenty-six of the 29 (89.7%) patients with rubella retinopathy had bilateral retinal disease. Twelve (2.2%) eyes were suspicious for rubella retinopathy and were given the designation of “peripheral stippling,” while 473 eyes (86.0%) had no evidence of rubella retinopathy.

Students with rubella retinopathy were much more likely to be hearing impaired; of the 29 students with rubella retinopathy, 28 (96.6%) had impaired hearing (*P* < 0.0001). Serologic testing suggested that positive rubella titers were associated with the presence of rubella retinopathy, with 55.1% of affected children testing positive (*P* = 0.001).

Ten eyes (1.8%) showed evidence of other ocular pathology such as ocular albinism (4), toxoplasmosis (1), macular scar (1), corneal scar (1), and retinal pigment atrophy. (1). One patient had undergone previous surgery for cataracts (this patient also had hearing impairment and rubella retinopathy), while no cases of congenital glaucoma were identified.

### 3.3. Congenital Rubella Syndrome Status

**Figure 3:**
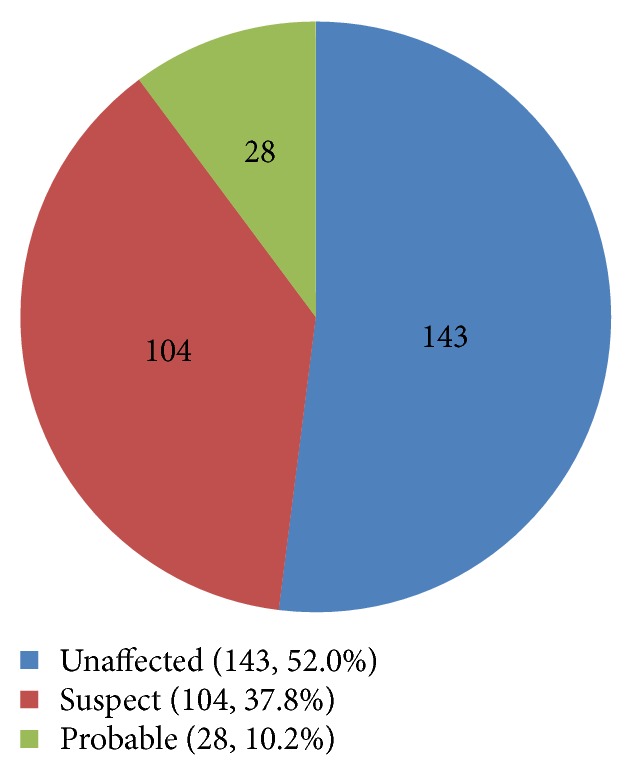
Numbers of cases of congenital rubella syndrome status based on revised Center for Disease Control guidelines.

CRS status was categorized based on modified Center for Disease Prevention and Control definitions (see Section 2.1, Methods) and was available for 275 participants. The majority of subjects (*n* = 143; 52.0%) were unaffected. There were 104 (37.8%) suspects and 28 (10.2%) probable cases of CRS (see Figure 3). Of the probable CRS cases, 57.1% (16) demonstrated positive rubella IgG, while 35.7% (10) were seronegative and 7.1% (2) did not have serology results.

## 4. Discussion

Congenital rubella syndrome is a major contributor to the global burden of preventable blindness and deafness. Cameroon currently offers immunization programs for both measles and mumps but does not cover rubella. The present study identified twenty-eight probable cases of CRS with clinical evidence in Northwest Cameroon.

The present study used revised CDC guidelines to determine the CRS status of patients. Hearing status was ascertained on history without being explicitly tested, and other probable etiologies of hearing impairment were not investigated. Moreover, serological criteria described in the CDC guidelines require the demonstration of rubella virus, rubella-specific IgM antibody, or infant rubella antibody levels that persist at a higher level and for a longer period of time than expected from passive maternal transfer of maternal antibodies in a population of infants. The cases described were older than could be tested for confirmatory serology; as a result, CRS probability was ascertained on clinical grounds.

This study found a significant relationship between rubella IgG seropositivity and hearing impairment in unvaccinated children. This relationship is confounded by the effect of age, as students with hearing impairment were significantly older than those without hearing impairment (*P* < 0.0001). If students with hearing impairment represented cases of CRS, then older age would be expected to underestimate the prevalence of IgG as diminution of antibodies is known to occur [16]. Conversely, prevalence of rubella IgG seropositivity has been shown to increase with age in unvaccinated populations from postnatal infection [17–19]. Without further testing, we cannot exclude the possibility that cases of children with hearing impairment and seropositivity for rubella IgG may have another etiology for hearing impairment, such as congenital CMV infection, and then acquired rubella postnatally. The substantially higher rate of seropositivity in the group with hearing impairment (48.8% versus 7.4%) is not fully explained by an age difference of 2.5 years between the groups. More likely is the possibility that many of the students with hearing impairment and positive serology represent unproven cases of CRS.

Despite the confounding effect of age, our results corroborate the findings of prior studies elsewhere. In a school for the deaf in Madras, India, rubella was the largest preventable cause of deafness, occurring in 29% of 374 cases [20]. In unvaccinated Bangladeshi children, a strong association between rubella antibody, clinical signs of intrauterine infection, and hearing impairment was reported [21]. Moreover, rubella antibody was detected in 74% of hearing-impaired children versus only 18% of those with normal hearing [21]. Taken together, these studies suggest that rubella is a significant contributor to the global burden of deafness and hearing impairment in unvaccinated populations.

Rubella retinopathy has a characteristic appearance but may mimic other “salt and pepper” retinopathies such as congenital syphilis, as well as other toxic or inherited diseases of the retinal pigment epithelium and the carrier state of X-linked ocular albinism (see Figure 1) [16, 22]. In the present study, rubella retinopathy was confirmed by a retina specialist to minimize errors in the clinical diagnosis. A similar study from Colombia examined 1383 institutionalized deaf people and identified salt and pepper retinopathy in 33.5% which was used to confirm prenatal rubella infection; these studies emphasize the role that ophthalmic findings can be used successfully in CRS screening programs [9].

The present study identified 29 cases of rubella retinopathy, of which 28 (96.6%) were in the hearing impaired population and 26 (89.7%) cases were bilateral. Our study is consistent with estimates in the literature reporting bilateral retinopathy in 80% of patients with CRS [16]. Only 55.1% of cases of rubella retinopathy demonstrated seropositivity for rubella IgG; however, other studies have observed a diminution of rubella IgG antibodies in children older than four years with documented rubella infection* in utero* [16].

Cataracts were expected to be observed in approximately one-third of CRS cases as reported in the literature [8, 23]; however, our study detected only one individual who had undergone previous cataract surgery, no individuals with significant cataracts, and no cases of congenital glaucoma. We believe that the low prevalence of cataract and congenital glaucoma found in this study could reflect an inherent sample bias in the study population. Unfortunately, due to limited teaching resources available in Cameroon, services for children suffering from both blindness and hearing impairment are not currently available at the schools that were studied.

WHO recommendations encourage all countries not routinely immunizing against rubella to quantify the burden of disease due to congenital rubella syndrome [24]. Interdisciplinary screening of infants for CRS should be widespread to enable early detection and treatment of complications. Women should be offered surveillance testing to identify those susceptible to rubella infection during gestation. Laboratories offering rubella serology, an essential component of reliable rubella surveillance, are unavailable throughout much of sub-Saharan Africa [25].

The present study provides clinical evidence of CRS in students in Northwest Cameroon using teleophthalmology. Moreover, a large population vulnerable to acquiring rubella infection later in life was identified: 92.6% of students with normal hearing were negative for rubella IgG. Other studies have estimated the rate of rubella serosusceptibility among women of reproductive age in West Africa to be 10% to 30% [26]. These results suggest that infection during gestation is possible in these vulnerable individuals, making future cases of CRS inevitable without intervention. We would strongly encourage the Government of Cameroon to consider augmenting its current mumps and measles vaccination programs with coverage for rubella. Such a program could reduce the rates of congenital and acquired rubella infection and lessen the burden of blindness, hearing impairment, and systemic morbidity.

## Figures and Tables

**Table 1:** Gestational age at time of confirmed rubella in pregnancy and clinical manifestations of congenital rubella syndrome [8].

Clinical manifestation	Gestational age (weeks)
Cataract	3–12
Congenital rubella retinopathy	2–18
Cardiac defects	3–13
Neurological defects	3–16
Deafness	2–18
Multiple defects	3–12
No defect	7–>20

Clinical manifestations of congenital rubella syndrome occur at specific points during gestation.
